# Health status in COPD cannot be measured by the St George's Respiratory Questionnaire alone: an evaluation of the underlying concepts of this questionnaire

**DOI:** 10.1186/1465-9921-11-98

**Published:** 2010-07-22

**Authors:** Leonie Daudey, Jeannette B Peters, Johan Molema, PN Richard Dekhuijzen, Judith B Prins, Yvonne F Heijdra, Jan H Vercoulen

**Affiliations:** 1 Department of Medical Psychology, Radboud University Nijmegen Medical Centre, Nijmegen, the Netherlands; 2 Department of Pulmonary Diseases, Radboud University Nijmegen Medical Centre, Groesbeek, the Netherlands

## Abstract

**Background:**

Improving patients' health status is one of the major goals in COPD treatment. Questionnaires could facilitate the guidance of patient-tailored disease management by exploring which aspects of health status are problematic, and which aspects are not. Health status consists of four main domains (physiological functioning, symptoms, functional impairment, and quality of life), and at least sixteen sub-domains. A prerequisite for patient-tailored treatment is a detailed assessment of all these sub-domains. Most questionnaires developed to measure health status consist of one or a few subscales and measure merely some aspects of health status. The question then rises which aspects of health status are measured by these instruments, and which aspects are not covered. As it is one of the most frequently used questionnaires in COPD, we evaluated which aspects of health status are measured and which aspects are not measured by the St George's Respiratory Questionnaire (SGRQ).

**Methods:**

One hundred and forty-six outpatients with COPD participated. Correlations were calculated between the three sections of the SGRQ and ten sub-domains of the Nijmegen Integral Assessment Framework, covering Symptoms, Functional Impairment, and Quality of Life. As the SGRQ was not expected to measure physiological functioning, we did not include this main domain in the statistical analyses. Pearson's r ≥ 0.70 was used as criterion for conceptual similarity.

**Results:**

The SGRQ sections *Symptoms *and *Total *showed conceptual similarity with the sub-domain Subjective Symptoms (main domain Symptoms). The sections *Activity, Impacts *and *Total *were conceptual similar to Subjective Impairment (main domain Functional Impairment). The SGRQ sections were not conceptual similar to other sub-domains of Symptoms, Functional Impairment, nor to any sub-domain of Quality of Life.

**Conclusions:**

The SGRQ could facilitate the guidance of disease management in COPD only partially. The SGRQ is appropriately only for measuring problems in the sub-domains Subjective Symptoms and Subjective Impairment, and not for measuring problems in other sub-domains of health status, such as Quality of Life.

## Background

COPD is a chronic and debilitating disease and a leading cause of morbidity and mortality worldwide [1]. According to the latest estimates of the World Health Organization (WHO), 210 million people have COPD and 3 million people died of COPD in 2005 [2]. Improving patients' health status is one of the major goals in COPD treatment [3].

Quality of life has become an important endpoint in medical care, but still there is no consensus on the definition of these concepts [4]. Smith and colleagues consider quality of life and health status to be separate constructs, in which quality of life is more related to mental health, whereas health status is more related to physical functioning [4]. The WHO uses a broader definition of health status, by defining health status as 'a state of complete physical, mental and social well-being, and not merely the absence of disease or infirmity'. Similarly, others [5,6] define health status as an overall concept covering physiological functioning, symptoms, functional impairment, quality of life, and social functioning as important main domains. These main domains were empirically found to be further divided into sixteen sub-domains [7,8], each sub-domain representing a unique aspect of health status. Despite differences in definitions found in the literature it has become clear that a patient's functioning consists of many conceptually distinct sub-domains. Patient-tailored treatment then requires assessment of all these sub-domains.

Questionnaires could facilitate the guidance of patient-tailored disease management by exploring which aspects of health status are problematic and which aspects are not. The past decade many questionnaires have been developed to measure health status. However, most of these instruments consists of only one or a few subscales and thus measure merely some aspects of health status. The question then rises which aspects of health status are measured by these instruments, and which aspects are not covered.

The St George's Respiratory Questionnaire (SGRQ), for instance, is one of the most frequently used and translated disease specific health status instruments in COPD [9-11]. A recent Pubmed search gave 555 hits (date 06/03/2010; terms SGRQ and St George's Respiratory Questionnaire). The SGRQ has been developed to allow comparative measurement of health between patient populations and to quantify changes in health following therapy [12]. The SGRQ consists of three sections and a total score: *Symptoms*, measuring the frequency and severity of respiratory symptoms; *Activity*, measuring limitation of activities by breathlessness and activities that cause breathlessness; *Impacts*, measuring disturbances in social and psychological functioning due to airway disease; *Total *score summarizes the impact of the disease on overall health status [12-14]. The SGRQ thus measures maximally three of the sixteen aspects of health status. It is not clear which aspects of health status are measured, and which aspects of health status are not measured by the SGRQ. This question is all the important to unravel, because the SGRQ, as many other questionnaires, is subject to conceptual confusion. The SGRQ initially was conceived as a standardized self-completed questionnaire for measuring health and perceived well-being ('QoL') in airways diseases [12]. In the literature, however, the SGRQ is interchangeably referred to as a measure of quality of life [15], health-related quality of life [16], health status [17], a measure for impaired health [18], or a measure of overall impact of the disease [19]. Different terms are used for the concept(s) the SGRQ measures. Additionally, since the SGRQ is often used as a criterion in validity testing of other instruments [20,21], it is essential to clarify which aspects of health status the SGRQ measures.

In the present study, we tested which aspects of health status are measured by the SGRQ in COPD, by comparing the SGRQ sections *Symptoms, Activity *and *Impacts *with multiple aspects of the health status domains Symptoms, Functional Impairment and Quality of Life.

## Material and methods

### Subjects

The 146 subjects took part on a longitudinal study on health status in COPD. Patients were recruited from three different outpatient centres in the Netherlands: Radboud University Nijmegen Medical Centre, Maas Hospital Boxmeer, and Rijnstate Hospital Arnhem. Patients had to fulfil the Global Initiative for Chronic Obstructive Lung Disease (GOLD) criteria of a post-bronchodilator FEV_1_% predicted between 30 and 80 percent with a reversibility of obstruction of less than 12% [1]. Patients suffering from primary co-morbidity or co-morbidity that prevented full adherence to the research protocol were excluded, as well as patients with an acute exacerbation, recent (<6 months) participation in a rehabilitation program, or who were not able to speak or read Dutch. One-hundred-and-sixty-eight patients participated in this study. After one year, the assessments were repeated in 146 patients (87% of included patients in first part). Reasons for dropout were diverse: passed away (N = 5), co-morbidity (N = 3), participation in a rehabilitation programme between the first and second assessments (N = 2), being too busy (N = 4), found participation too exhausting (N = 3), or no transportation (N = 2). For three patients the reasons for dropout were unknown. Data of these 146 patients were used in the present study. The inclusion procedure is described in more detail elsewhere [7]. The study was approved by the Medical Ethics Committee CMO Region Arnhem-Nijmegen (P02.1411L; CMO-nr 2002/047). Subjects gave informed consent.

### Procedures

Subjects visited the Department of Pulmonary Diseases twice. Physiological assessments were performed and subjects received the Aktometer (accelerometer measuring actual physical activity) [22]. Two weeks later subjects completed questionnaires by the TestOrganiser, a computer program developed by the Department of Medical Psychology and the Department of Instrumental Services of the Radboud University Nijmegen Medical Centre [7]. Questionnaires were presented in the same layout as the paper-and-pencil versions, and a simple response board enabled subjects with no prior computer experience to operate the TestOrganiser easily.

### Measurements

Demographic data were recorded. Pulmonary function tests were performed, including transfer capacity for carbon monoxide using the Jaeger masterlab-spirometer according to ERS-criteria [23], and indices of body composition (BodyStat 1997).

### St George's Respiratory Questionnaire (SGRQ)

The SGRQ consists of 50 items with weighted responses divided in three sections - *Symptoms*, *Activity*, and *Impacts *- and a *Total *score [12-14]. Scores are expressed as percentages of the maximally possible sum of weights. A score of zero represents no health impairment, a score of 100 means maximal health impairment.

### Health status main domains Symptoms, Functional Impairment, and Quality of Life

Health status was measured by the Nijmegen Integral Assessment Framework (NIAF) [7]. The NIAF provides a detailed and empirical definition of health status and covers the domains Physiological Functioning, Symptoms, Functional Impairment, and Quality of Life. These four main domains were found to be subdivided into 15 distinct sub-domains [7]. In another study [8], we found that fatigue was an additional sub-domain. Factor analyses were used to identify underlying concepts in the data. Social Functioning did not emerge as a separate factor, aspects of social functioning were part of the main domains Quality of Life and Functional Impairment. The sub-domains are measured by different existing instruments, and for each sub-domain a Sub-domain Total Score (STS) was calculated. As the SGRQ was not expected to measure physiological functioning, in this study we only evaluated the ten sub-domains of the main domains Symptoms, Functional Impairment, and Quality of Life. See Table 1 for definitions of the sub-domains and corresponding instruments.

**Table 1:** Main domains Symptoms, Functional Impairment and Quality of Life of the Nijmegen Integral Assessment Framework

Sub-domain	Definition	Instrument (subscales)
**Symptoms**		
Subjective Symptoms	The patient's overall burden of pulmonary symptoms	PARS-D: Global Dyspnea Activity, Global Dyspnea Burden, Dyspnea Activity [7]; QoLRiQ: Breathing Problems [33]
Dyspnea Emotions	The level of frustration, depressive feelings, and anxiety a person experiences when dyspnoeic	DEQ: Frustration, Mood, Anxiety [7]
Expected Dyspnea	The level of dyspnea that a patients expect to experience during specific activities no longer performed	PARS-D: Expected Dyspnea [7]
Fatigue	The level of experienced fatigue	CIS: Subjective fatigue [34]

**Functional Impairment**		
Actual Physical Activity	The actual physical activity a patient performs during two weeks	Aktometer (electronic accelerometer) [22]
Behavioral Impairment	The extent to which a person cannot perform specific and concrete activities as a result of having the disease	SIP: Body Care & Movement, Home Management, Mobility, Ambulation [35]
Subjective Impairment	The experienced degree of impairment in general, and in social functioning	QoLRiQ: General Activities, Social Activities [33]; Global Impairment [7]; SIP: Social Interaction, Burden [35]

**Quality of Life**		
General Quality of Life	Mood, anxiety, and the satisfaction of a person with his/her life as a whole	Satisfaction With Life Scale [36] Symptom Check List: Anxiety [37] BDI: Primary Care [38]
Health-related Quality of Life	Satisfaction related to physiological functioning and the future	Satisfaction Physiological Functioning, Satisfaction Future [7]
Satisfaction Relations	Satisfaction with the (absent) relationships with spouse and others	Satisfaction Spouse, Satisfaction Social [7]

PARS-D: Physical Activity Rating Scale-Dyspnea; QoLRiQ: Quality of Life for Respiratory Illness Questionnaire; DEQ: Dyspnea Emotions Questionnaire; CIS: Checklist Individual Strength; SIP: Sickness Impact Profile; BDI, Beck Depression Inventory

### Statistical Analyses

The relationships between the sections of the SGRQ and the sub-domains of the NIAF, as well as the intercorrelations of the SGRQ sections, were analyzed by Pearson correlation coefficients. To avoid Type I error due to multiple testing P was set at 0.01. A Pearson's r ≥ 0.70 was used as criterion for conceptual similarity between the sections of the SGRQ and the sub-domains of the NIAF [24].

## Results

### Subjects

The study sample could be characterized as predominantly male, low educated, and living with a partner (Table 2). Most subjects were GOLD II/III patients. Some subjects were classified in GOLD I or IV, due to normal variation in FEV_1 _between the time of the first assessment and second assessment one year later.

**Table 2:** Demographic, clinical data, and data of the St George's Respiratory Questionnaire of participating COPD patients

Variable	Mean ± SD
Male sex %	76.7
Age (years)	65.8 ± 9.0
Education %	
Low	48.6
Middle	29.5
High	19.9
Personal situation %	
Partner	77.8
Divorced	6.3
Widowhood	8.3
Single	7.6
Cigarette smoking %	
Current	41.8
Former	45.9
Never	11.0
BMI (kg/m^2^)	25.9 ± 4.1
FEV_1 _(L)	1.6 ± 0.5
FEV_1 _% predicted	53.6 ± 13.9
FEV_1_/FVC %	44.0 ± 11.4
TLC % predicted	103.7 ± 14.6
RV % predicted	128.3 ± 30.3
TL_CO _% predicted	62.3 ± 21.5
GOLD %	
I	2.1
II	58.9
III	34.2
IV	4.8
SGRQ section	
Symptoms	40.9 ± 24.8
Activity	40.9 ± 21.8
Impacts	20.2 ± 13.5
Total	30.2 ± 15.4

Data are presented as mean ± SD unless otherwise indicated. Percentages may not add up to 100 due to missing data (three patients with no specified education, two patients with no specified smoking habits). BMI: body mass index; FEV_1 _% predicted: forced expiratory volume in one second as percentage predicted; FEV_1_/FVC %: forced expiratory volume/forced vital capacity; TLC: total lung capacity; TLC % predicted: total lung capacity as percentage predicted; RV: residual volume; RV: residual volume as percentage predicted; TL_CO _% predicted: transfer capacity (of lung) for carbon monoxide as percentage predicted; GOLD: Global Initiative for Chronic Obstructive Lung Disease; SGRQ: St George's Respiratory Questionnaire.

### Conceptual similarity between sections of the SGRQ and sub-domains of the NIAF

The SGRQ sections were significantly correlated to many health status aspects, however conceptual similarity (r ≥ 0.70) was only reached for two sub-domains of the NIAF (Table 3). The SGRQ sections *Symptoms *and *Total *were conceptual similar to the NIAF sub-domain Subjective Symptoms (main domain Symptoms). The SGRQ sections *Activity, Impacts*, and *Total *were conceptually similar to the NIAF sub-domain Subjective Impairment (main domain Functional Impairment).

**Table 3:** Correlations between the St George's Respiratory Questionnaire and the Nijmegen Integral Assessment Framework^#^

	St George's Respiratory Questionnaire			
	**Symptoms**	**Activity**	**Impacts**	**Total**
**Nijmegen Integral Assessment Framework**				
**Symptoms**				
Subjective Symptoms	0.70^¶^	0.64	0.60	0.74^¶^
Dyspnea Emotions	0.25	0.31	0.32	0.35
Dyspnea Expected	0.43	0.59	0.43	0.57
Fatigue	0.47	0.57	0.60	0.65
**Functional Impairment**				
Actual Physical Activity	---	0.42	0.31	0.34
Behavioral Impairment	0.28	0.65	0.54	0.61
Subjective Impairment	0.67	0.70	0.71	0.81
**Quality of Life**				
General Quality of Life	0.50	0.46	0.52	0.57
Health-related Quality of Life	0.43	0.42	0.46	0.51
Satisfaction Relations	0.24	---	---	0.21

^#^only significant correlations (p < 0.01) are shown; ^¶^Pearson's r ≥ 0.70 (criterion for conceptual similarity)

### Intercorrelations of the SGRQ sections

Intercorrelations between the SGRQ sections were moderate to high (Table 4). The SGRQ section *Total *exceeded the criterion of conceptual similarity with all SGRQ sections (r ≥ 0.70, p < 0.01). The correlation between the sections *Impacts *and *Activity *almost reached the criterion of conceptual similarity (r = 0.69, p < 0.01).

**Table 4:** Intercorrelations between sections of the St George's Respiratory Questionnaire^#^

	St George's Respiratory Questionnaire			
	**Symptoms**	**Activity**	**Impacts**	**Total**

**Symptoms**	1.00	--	--	--
**Activity**	0.50	1.00	--	--
**Impacts**	0.54	0.69	1.00	--
**Total**	0.73^¶^	0.88^¶^	0.91^¶^	1.00

^#^only significant correlations (p < 0.01) are shown; ^¶^Pearson's r ≥ 0.70 (criterion for conceptual similarity)

## Discussion

The present study evaluated which aspects of health status are measured by the sections of the SGRQ, and which aspects of health status are not covered by the SGRQ.

The sections of the SGRQ correlated significantly with most sub-domains of the NIAF, indicating that the SGRQ was related to many health status aspects. However, most correlations were low to moderate and well below 0.70, indicating that shared variance was too low to conclude that sections of the SGRQ were conceptually similar to these sub-domains.

Applying the criterion of conceptual similarity, the SGRQ measured two of the ten evaluated sub-domains of health status. The SGRQ sections *Symptoms *and *Total *showed conceptual similarity with the sub-domain Subjective Symptoms (main domain Symptoms), the SGRQ sections *Activity, Impacts*, and *Total *showed conceptual similarity with the sub-domain Subjective Impairment (main domain Functional Impairment).

In a previous study [7] we found a high correlation between the sub-domains Subjective Impairment and Subjective Symptoms. The instruments included in these sub-domains were different with respect to the content of the items, but had in common that the item-and-response format required highly subjective and general interpretations by the patient. It was argued that both sub-domains measured highly subjective notions of 'being ill', also referred to as illness perceptions [25]. As the SGRQ reached the criterion for conceptual similarity with these two sub-domains, this would imply that the SGRQ in fact measures illness perceptions, related to symptoms (section *Symptoms *and *Total*) and functional impairment (sections *Activity *and *Impacts*). This conclusion is underlined by the high intercorrelations between the SGRQ sections, some correlations even exceeding the criterion for conceptual similarity.

Although illness perceptions related to symptoms and functional impairment are very relevant concepts, many other important aspects of health status are not covered by the SGRQ. With respect to the SGRQ as a measure of aspects of symptoms, these are restricted to the subjectively experienced severity of pulmonary symptoms. Other important aspects of symptoms, such as dyspnea-related emotions, are not measured specifically. With respect to functional impairment, only the subjectively experienced impairments are measured by the SGRQ. Impairment on the behavioural level or actual physical activity level is not measured by the SGRQ sections. Furthermore, the present study showed that the SGRQ does not measure any of the three sub-domains of quality of life evaluated in this study (General Quality of Life, Health-related Quality of Life, and Satisfaction Relation). Finally, since the SGRQ measures merely two sub-domains of the ten evaluated sub-domains, the SGRQ does not provide a detailed measurement of health status. Similarly, present data show that the SGRQ should be considered a valid measure of impaired health in COPD, as the SGRQ originally was conceived. However, the SGRQ measures only two aspects of impaired health (subjective symptoms and subjective impairment). To measure all aspects of impaired health, and thereby allowing patient-tailored treatment, other instruments need to be included as well.

Some methodological issues need to be addressed. First, the NIAF is not the definite answer to the problem of conceptual confusion in current health status instruments. Other aspects of health status not included in the framework may be relevant to COPD patients. This needs to be addressed in future studies, in which patient feedback should be incorporated. Nevertheless, this framework does provide a much more detailed definition of health status, as expressed by the many sub-domains, and is much more formulated in terms of empirical observations than found in the literature. Each sub-domain represents a (conceptually) unique health status aspect. At least 16 sub-domains are measured to provide a detailed picture of the health status of a COPD patient.

Second, using the criterion of conceptual similarity (r ≥ 0.70) as a standard for validity seems a very strict criterion. However, considering the conceptual confusion in health status, one must be carefully interpreting results of earlier validity studies. Often, much lower correlations are accepted as evidence for the validity of the instrument under scrutiny. For example, a correlation between two instruments of 0.40 may be statistically significant, but it indicates only 16% of shared variance. Unambiguous conclusions concerning conceptual similarity between two instruments can only be drawn from the results using a strict approach.

The present study focuses on the relationships between the SGRQ sections and the main domains Symptoms, Functional Impairment, and Quality of Life. Therefore, the conclusions of the present study are not applicable with respect to physiological functioning. However, from a theoretical point of view it is unlikely that a questionnaire will provide a direct measure of physiological processes. For example, studies to date [26,27] often show a relationship between FEV_1 _and the SGRQ. However, these correlations are low to moderate and do not exceed the criterion of conceptual similarity.

With respect to generalizability of the present study, we believe that the present sample may be an adequate reflection of a the Dutch population of patients with COPD seen in an outpatient clinic. This sample may however not be representative for subgroups of COPD such as patients in pulmonary rehabilitation or patients with primary co-morbidity, which were two major exclusion criteria.

An important clinical implication of the present study is that the SGRQ could facilitate the guidance of disease management only partially. The SGRQ can only be used appropriately for exploring problems in the sub-domains Subjective Symptoms and Subjective Impairment, and not for exploring problems in other sub-domains of health status, such as aspects of quality of life.

Most instruments claiming to measure specific aspects of health status contain only two to five subscales. Thus, at best only some aspects of health status are measured by a specific instrument. This not only has implications for clinical practice, but also for research purposes. In pharmacological trials, the drug under study may have beneficial effects on some aspects of health status, but not on other aspects. If the instruments used measure only few aspects of health status beneficial effects may be missed. With respect to the use of instruments in clinical practice, the present results indicate that one single instrument cannot provide sufficient information on a patient's health status to effectively tailor treatment to the needs of the individual patient, since measuring all aspects of health status is a prerequisite for patient-tailored treatment. This requires combining different instruments into a battery of instruments measuring multiple aspects of health status. However, implementing instruments in daily practice to facilitate disease management requires that instruments are not too time consuming. The past decade a few short instruments have been developed specifically to allow measurement of health status aspects in routine care, such as the Clinical COPD Questionnaire [28], the Respiratory Illness Questionnaire-monitoring 10 [29], and the EuroQoL [30]. None of these instruments provide a detailed picture of a patient's health status. Recently, we developed the Nijmegen Clinical Screening Instrument (NCSI), an instrument which can be used in routine care [31]. The NCSI is based on the NIAF and measures eleven sub-domains of physiological functioning, symptoms, functional impairment, and quality of life. The NCSI enables a quick (15-25 minutes) and detailed assessment of health status. Also, the COPD Assessment Test (CAT) was developed [32], 'a validated short and simple instrument for assessing the impact of COPD on health status'. The CAT is constructed as a uni-dimensional instrument, i.e. measuring one single concept, as expressed in a single score. In addition, the correlation between the CAT and the SGRQ-C was well above the criterion for conceptual similarity (r = 0.80) [32]. Taken together, it is very likely that the CAT, like the SGRQ, measures illness perceptions. How important illness perceptions may be, patient-tailored treatment requires a detailed assessment of many aspects of health status. Therefore, the CAT also will have limited value in patient-tailored treatment.

## Conclusions

Detailed measurement of health status in patients with COPD is a prerequisite for patient-tailored treatment. However, carefulness should be noted when selecting instruments to measure health status, because most instruments measure only a few aspects of health status. The SGRQ can only be used appropriately for measuring problems in the sub-domains Subjective Symptoms and Subjective Impairment, and not for measuring problems in other sub-domains of health status, such as aspects of Quality of Life. Different instruments should be combined to provide a detailed picture of a patient's health status.

## Competing interests

The authors declare that they have no competing interests.

## Authors' contributions

LD participated in the design of the study, the acquisition of the data, performed statistical analyses and interpreted the data, and drafted the manuscript. JBPe participated in the acquisition of the data, and in the critical revision of the manuscript for important intellectual content. JM participated in the design of the study, the acquisition of the data, and in the critical revision of the manuscript for important intellectual content. PNRD participated in the critical revision of the manuscript for important intellectual content. JBPr participated in the critical revision of the manuscript for important intellectual content. YFH participated in the acquisition of the data, and the critical revision of the manuscript for important intellectual content. JHV conceived the study, participated in its design and coordination and helped to draft the manuscript. All authors read and approved the final manuscript.

## Acknowledgements

We are indebted to Dr. F. van den Elshout (pulmonologist, Rijnstate Hospital, Arnhem) and Dr. R. Bunnik (pulmonologist, Maas Hospital, Boxmeer) for their contribution in the patient recruitment and the multidisciplinary Taskforce Assessment of the Department of Pulmonary Rehabilitation for their invaluable contributions to the development of the conceptual models.

The study was supported by grants of the Dutch Asthma Foundation and GlaxoSmithKline.
